# Analysis of α-synuclein levels related to LRRK2 kinase activity: from substantia nigra to urine of patients with Parkinson’s disease

**DOI:** 10.1080/19768354.2021.1883735

**Published:** 2021-02-17

**Authors:** Daleum Nam, Ami Kim, Sun Jung Han, Sung-Ik Lee, Sung-Hye Park, Wongi Seol, Ilhong Son, Dong Hwan Ho

**Affiliations:** a InAm Neuroscience Research Center, Gunpo, Republic of Korea; b Department of Neurology, Sanbon Medical Center, College of Medicine, Wonkwang University, Gunpo-si, Republic of Korea; c Department of pathology, Seoul National University College of Medicine, Seoul National University Hospital, Seoul, Republic of Korea

**Keywords:** Parkinson’s disease, α-synuclein, leucine-rich repeat kinase 2, ELISA

## Abstract

Research on Parkinson’s disease (PD) has been focused on the development of PD diagnostic tools as much as the development of PD therapeutics. Several genetic culprits of PD, including DJ-1, Leucine-rich repeat kinase 2 (LRRK2), and α-synuclein (α-syn), have been investigated as markers of PD in human biofluids. Unfortunately, the approaches to develop PD diagnostic tools are impractical, and there is a considerable demand for an appropriate marker of PD. The measurement of α-syn in biofluids has recently been made more accurate by examining monomers and aggregates separately using enzyme-linked immunosorbent assay (ELISA). Previously, we reported on the development of two types of sandwich ELISA for total α-syn and MJFR-14-6-4-2 antibody-specific α-syn fibrillar oligomers. The pathogenic LRRK2 G2019S mutation is related to increased α-syn secretion in the extracellular space. We tested our established ELISA using differentiated SH-SH5Y cells transfected with LRRK2 G2019S. The secretory levels of fibrillar oligomeric α-syn divided by total α-syn were significantly increased in LRRK2 G2019S-expressing cells. Additionally, substantia nigra lysates or concentrated urine from PD patients and non-PD subjects were analyzed. We observed ambiguous changes in the levels of total or fibrillar oligomeric α-syn and their ratio between PD and non-PD. Despite the insignificant increase in the relative levels of fibrillar oligomeric α-syn to total α-syn in PD, the duration of disease progression after diagnosis significantly corresponded to the relative levels of fibrillar oligomeric α-syn to total α-syn in the urine. These results might provide greater understanding for the next stage of development of α-syn ELISAs.

## Introduction

Parkinson’s disease (PD) is the most common neurodegenerative movement disorder worldwide. The patients with PD suffered with cruel motor manifestations, such as resting tremor, rigidity, and bradykinesia, from early stage of disease progression until death (Beitz 2014). Diagnosis of PD has relied on the expression or severity of motor symptoms, which is signified by the matured progression of PD (Opara et al. 2017). Thus, identifying prognostic markers in PD patients could provide critical assistance for clinical treatments such as L-DOPA, selegiline, or tolcapone (Brannan and Yahr 1995; Davis et al. 1995; Adamiak et al. 2010). Currently, several genes have been reported to be associated with the development of PD (Klein and Westenberger 2012). α-Synuclein (α-syn) is a major component of Lewy bodies and is considered to be a pathological marker of PD (Kim et al. 2014). Indeed, the aggregation of α-syn in dopaminergic neurons as well as its secretion into the extracellular space would be one of the pathomechanisms of PD progression (Marques and Outeiro 2012). For this reason, it is speculated that α-syn could be a feasible PD diagnostic marker through its measurement in biofluids, including cerebrospinal fluids, blood, saliva, and tears (Atik et al. 2016). In addition, we previously reported the presence of urinary α-syn, and a distinct change between the fibrillar α-syn oligomer and the spherical α-syn oligomer which were detected by the MJFR-14-6-4-2 antibody and ASyO5 antibody, respectively, in the urine of PD patients (Nam et al. 2020).

Numerous studies have revealed that the release of α-syn is affected by PD-related genes, and the G2019S mutation of LRRK2 has been shown to be responsible for the increase in α-syn secretion (Bae et al. 2018; Schapansky et al. 2018; Bieri et al. 2019). Since the age-associated penetrance of G2019S LRRK2 in idiopathic PD has been reported in previous studies (Marder et al. 2015; Lee et al. 2017), we hypothesized that the presence of the G2019S LRRK2 mutation in dopaminergic neurons could harbor a comparable condition of idiopathic PD and its release of α-syn. Herein, we measured and compared the intracellular and extracellular levels of fibrillar α-syn along with their total amount of α-syn in dopaminergic neuron-like cells with or without G2019S expression. Furthermore, the levels of fibrillar α-syn oligomer and total α-syn in the substantia nigra and urine samples were compared between PD patients and the non-PD subjects.

## Materials and methods

### Cell culture and transfection

The human neuroblastoma cell line, SH-SY5Y, was used for the cell-based system following ectopic expression of LRRK2 G2019S mutants. SH-SY5Y cells were differentiated with all-trans retinoic acid (10 μM) for 7 days to induce dopaminergic neuron-like cells. On the last day of differentiation, the differentiated SH-SY5Y cells (dSH) were transfected with 1.2 μg of DNA plasmid containing G2019S LRRK2 or vector control for 48 h using Lipofectamine LTX & PLUS reagent (Invitrogen, 15338-100). The culture media was collected and centrifuged at 2,000 rpm for 10 min at 4°C to remove the cell debris. The supernatants were concentrated 5-fold that of the original culture media using a 10 K filter. Cells were harvested with 100 μl of lysis buffer (PBS with 1% Triton X-100, 1× Xpert protease inhibitor cocktail solution, and 2 mM EDTA) and centrifuged under the same condition as culture medium collection. Following centrifugation, 80% of the supernatants were transferred to a new tube for ELISA and scaled up with 420 μl of lysis buffer. The remaining 20% of the supernatants were mixed with 5× sample buffer to make the final 1× lysate mixtures. Finally, samples were sonicated and subjected to 95°C for 5 min for their use in western blot assay.

### Sandwich ELISA

Our previous study demonstrated the establishment of sandwich ELISA for total α-syn (Total-αS) or fibrillar oligomer α-syn (Fila-αS) using Anti-α-synuclein antibody/Clone #42 (BD bioscience, 610786) or Anti-α-synuclein filament antibody [MJFR-14-6-4-2]-Conformation-Specific (Abcam, ab209538), respectively. The rest of the ELISA procedure was performed under the same condition as our previous study (Nam et al. 2020).

### Human substantia nigra lysates

Donated brain tissues from non-PD or PD patients were obtained from the SNUH Brain Bank (Seoul, Korea), and their clinical summary is described in Table 1. Tissues were lysed with M-PER™ Mammalian Protein Extraction Reagent (Thermo, 78503) and homogenated using Kontes™ Pellet Pestle Motor (Sigma, Z359971). Following centrifugation at 2,000 rpm for 10 min at 4°C, the supernatants were mixed with 5× sample buffer for the western blot assay or collected for ELISA analysis.

**Table 1.** Clinical information of donated human brain tissues.

	Ctrl			PD		
	1	2	3	1	2	3
	BB19-5	BB19-11	BB18-9	BB18-10	BB17-10	BB17-16
Sex	M	M	M	F	M	M
Age	61	87	55	74	69	79
Postmortem interval (h)	12.5	9	9	19	6	4
Clinical Diagnosis	Stomach-carcinoma	Cholangio-carcinoma	Hypoparynx cancer, diabetes mellitus	PD	PD	PD
Braak Stage	I/VI^a^	I/VI^a^	0/VI^b^	I/II	I	I
NIA-AA ABC Score	NA	NA	NA	LOW	LOW	LOW

^a^ Only mild pTau/p62 positive neuropil threads in entorhinal cortex; ^b^Only mild pTau positive neuropil threads in entorhinal cortex and amygdala; NA: not applicable.

### Clinical process of urine collection and concentration

The processes of urine collection and analysis were approved by the Institutional Review Board of Sanbon Medical Center, Wonkwang University (IRB2013-24). Based on the UK brain criteria (Daniel and Lees 1993), neurological experts diagnosed PD patients and prescribed the L-DOPA dosage. All PD patients volunteered for urine collection and informed consent was signed by either the patients or their representatives. Non-PD urine was donated by hospitalized patients or their deputies in the family relationship at the Sanbon Medical Center. Non-PD subjects agreed to participation in the study and signed the consent form. We excluded urine specimens with proteinuria, hematuria, and glycosuria. Eventually, urines from 13 PD patients and 8 non-PD subjects were selected for this study (Table 2). Urine samples were mixed with 1 mM phenylmethyl-sulfonyl fluoride (PMSF), 2 mM EDTA, 1 mM leupeptin, and 1% Triton X-100 and incubated for 30 min on ice. The mixtures were centrifuged at 10,000 g for 30 min at 4°C to discard the salt crystal or protein adducts/aggregates. We concentrated 1.5 ml of supernatants to 300 μl. The concentrated urines were dialyzed twice with an identical volume of PBS, and 100 μl of samples were used for the respective ELISA analyses.

**Table 2.** Summary of human urine samples.

	Age	Sex	Duration (years)	L-DOPA dose (mg)
non-PD	72.75 (±9.16)	F = 6, M = 2 (*n* = 8)	NA	NA
PD	72.85 (±6.48)	F = 7, M = 6 (*n* = 13)	7.77 (±2.17)	130.77 (±138.13)

NA: not applicable.

### Western blot analysis

Samples were loaded on 4–20% Mini-PROTEAN® TGX™ Precast Protein Gels, 15-well, 15 µl (Bio-rad, #4561096), then proteins were transferred onto Amersham™ Protran™ NC Nitrocellulose Membranes (GE Healthcare, 45-004-001). Membranes were blocked by 1% bovine serum albumin in Tris-buffered saline with 0.1% Tween 20 (TBST). The immunoreaction enhancer solution Can Get Signal™ (TOYOBO, NKB-101) was used for the application of the primary and secondary antibodies listed in Table 3.

**Table 3.** Summary of used antibodies for western blot.

Antibody name	Dilution
Recombinant Anti-LRRK2 (phospho S1292) rabbit monoclonal antibody [MJFR-19-7-8] (abcam, sb203181)	1:500
Anti-c-Myc mouse monoclonal antibody [9E10] (Santa Cruz, sc-40)	1:2000
Anti-LRRK2/Dardarin mouse monoclonal antibody [N241A/34] (Neuromab, 75-253)	1:1000
Anti-β-actin mouse monoclonal antibody [C4] (Santa Cruz. Sc-47778)	1:4000
Anti-α-tubulin mouse monoclonal antibody [B-5-1-2] (Sigma-aldrich, T5168)	1:10000
Peroxidase-conjugated AffiniPure Goat Anti-Mouse IgG (H + L) (#115-035-003; Jackson Immunoresearch Laboratories Inc.)	1:5000
Peroxidase-conjugated AffiniPure Goat Anti-Rabbit IgG (H + L) (#111-035-144; Jackson Immunoresearch Laboratories Inc.).	1:5000

### Data analysis and statistics

The ELISA absorbances were measured with Synergy 2 (Biotek) and analyzed using Prism 8 (GraphPad). Multi Gauge (Fujifilm) was used to estimate the density of detected proteins. Data are represented as mean ± SEM in all diagrams. Statistical significance is indicated as follows: *p < 0.05, **p < 0.01, ***p < 0.001, ****p < 0.0001.

## Result

### Expression of G2019S LRRK2 in dopaminergic neurons promotes the release of fibrillar α-syn oligomers

**Figure 1.**
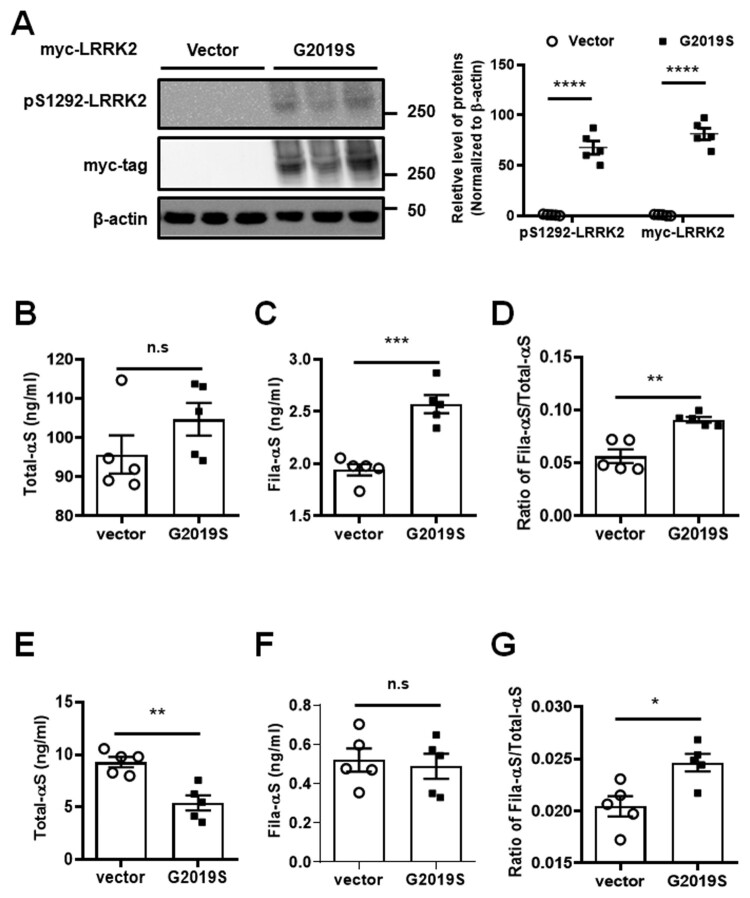
Increases in fibrillar α-syn oligomer by the ectopic expression of G2019S LRRK2. (A) Expression of myc-tagged G2019S LRRK2 (myc-tag) and its autophosphorylation on the S1292 site (pS1292-LRRK2) analyzed with western blot. The measured intensity of protein bands normalized with β-actin levels and presented. Two-way ANOVA with Tukey post hoc test applied (*n* = 5). The lysates of cells transfected with vector or G2019S analyzed using ELISAs for Total-αS (B) and Fila-αS (C), and Fila-αS levels normalized with Total-αS are presented as the ratio of Fila-αS/Total-αS (D). Concentrates of culture media subjected to ELISAs of Total-αS (E) and Fila-αS (F), and the ratio of Fila-αS levels divided by Total-αS (G) are estimated. Student’s *t*-test was used for statistical analysis (*n* = 5).

Previous reports demonstrated that the ectopic expression of G2019S LRRK2 enhanced the secretion of α-syn into the extracellular space (Bae et al. 2018; Schapansky et al. 2018; Bieri et al. 2019). To investigate the proportion of α-syn in the intra- and extracellular space relative to the expression of G2019S LRRK2, we respectively introduced vector or myc-tagged G2019S LRRK2 plasmid in dSH (G2019S-dSH) for 48 h and confirmed autophosphorylation on the S1292 site of LRRK2 and myc-tag expression (Figure 1(A)). We observed a significant elevation of Fila-αS levels and the ratio of Fila-αS/Total-αS from G2019S-dSH lysates, but Total-αS levels was not (Figure 1(B–D)). Meanwhile, the released Total-αS in G2019S-dSH was significantly lower than that in the vector control, along with similar levels of Fila-αS in the extracellular space between the vector and G2019S-dSH (Figure 1(E,F)). The ratio of Fila-αS/Total-αS showed a significant increase in G2019S-dSH (Figure 1(G)). From these results, we can conclude that the expression of G2019S LRRK2 relatively enhances the release of fibrillar α-syn oligomer in dopaminergic neurons.

### Decrease in the ratio of fibrillar α-syn oligomer/total α-syn along with the increased LRRK2 activity in the human substantia nigra

**Figure 2.**
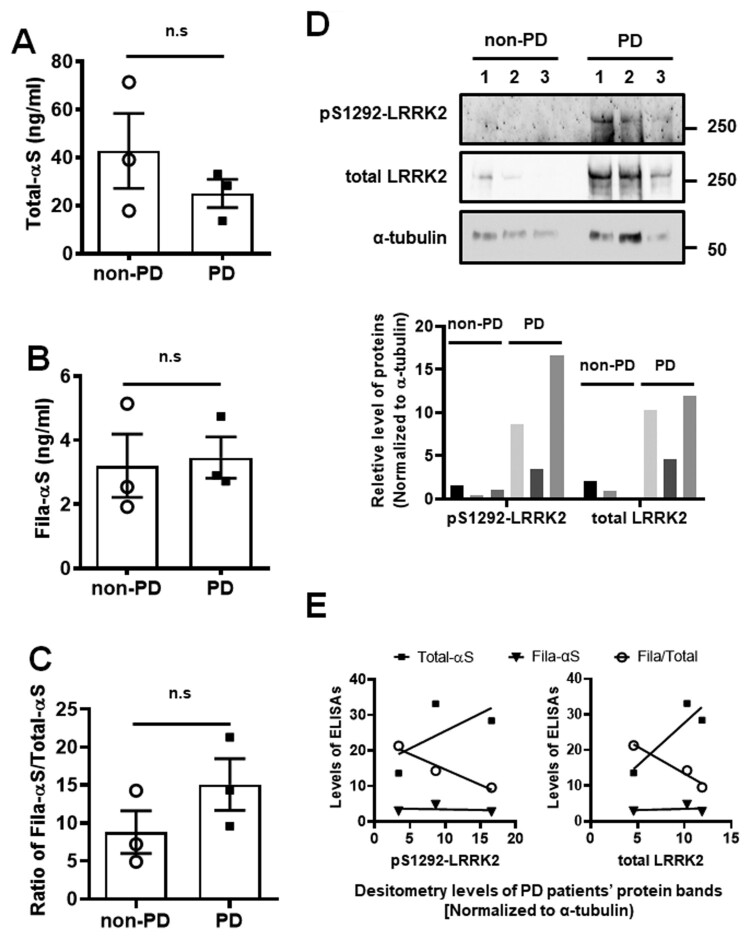
Analyses of α-syn along with LRRK2 in human brain SN tissues from PD patients and non-PD subjects. The lysates of SN tissues analyzed using the ELISA of Total-αS (A) and Fila-αS (B), and the ratio of Fila-αS/Total-αS (C) is estimated. Student’s *t*-test used for statistical analysis (*n* = 3). (D) For western blot, 20 µl of ELISA samples used, and the levels of total LRRK2 and pS1292-LRRK2 shown. (E) The levels of pS1292-LRRK2 or total LRRK2 of PD patients compared with the Total-αS (square), Fila-αS (opened circle), and the ratio of Fila-αS/Total-αS (reversed triangle). X-axis represents the densitometry levels of PD patients’ pS1292-LRRK2 and total LRRK2 protein bands. The correlation curve was interpolated using a linear standard curve and presented without the 95% confidence bands of the best-fit line.

To compare the LRRK2 levels along with various α-syn levels in the human substantia nigra (SN), we analyzed three different SN tissues from non-PD subjects and PD patients using ELISA and western blot assays. The levels of Total- or Fila-αS, and the ratio of Fila-αS/Total-αS were not significantly different between non-PD and PD SN lysates (Figure 2(A–C)). However, the three-PD patients showed a dramatic increase in LRRK2 autophosphorylation and total LRRK2 levels compared with non-PD subjects (Figure 2(D)). Interestingly, the Total-αS levels of PD samples increased along with the levels of LRRK2 autophosphorylation or total LRRK2, but Fila-αS levels were not changed by LRRK2. Therefore, the ratio of Fila-αS/Total-αS was decreased along with the pS1292- or total LRRK2 (Figure 2(E)). Because pS1292-LRRK2 of non-PD tissues and one non-PD patient’s LRRK2 levels were undetectable, the comparison between pS1292- or total LRRK2 and α-syn levels in non-PD could not be analyzed. Taken together, up-regulation of LRRK2 kinase activity in PD might responsible for the accumulation of total α-syn in dopaminergic neurons.

### Analyses of PD-related parameters along with urinary α-syn levels

**Figure 3.**
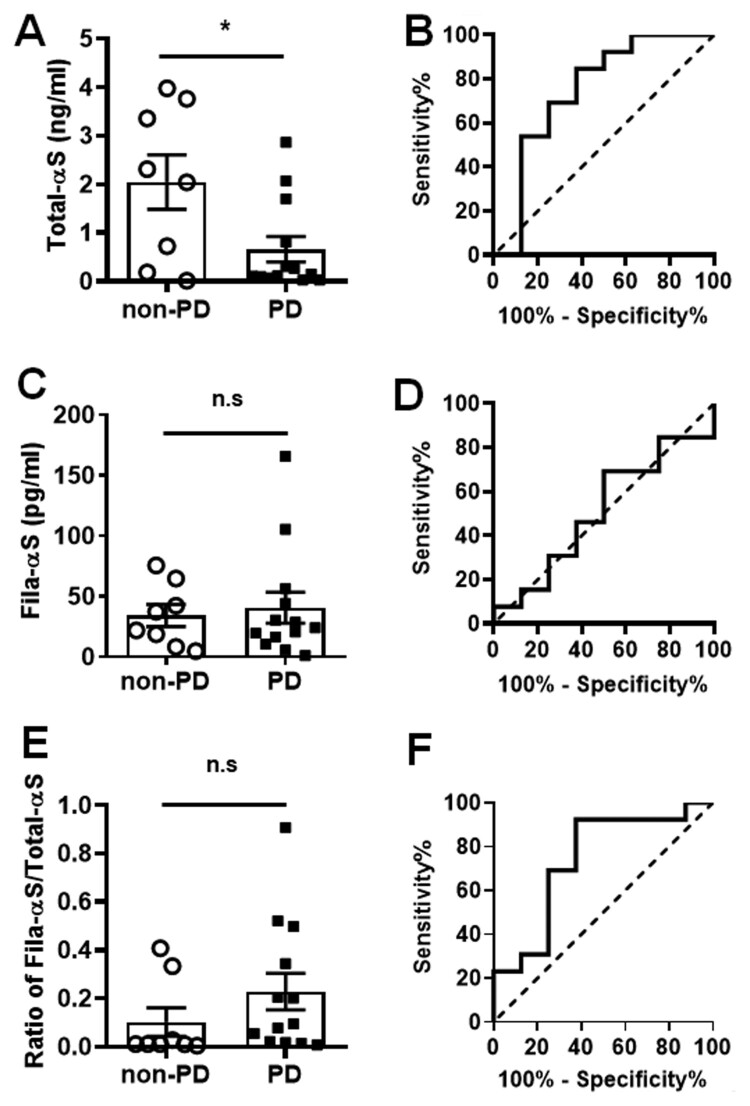
Quantification of urinary total α-syn and fibrillar oligomer α-syn. Urine samples from non-PD and PD patients was subjected to Total-αS (A) or Fila-αS (C), and the ratio of Fila-αS/Total- αS was estimated (E). The ROC curves for Total-αS (AUC = 0.75) (B), Fila-αS (AUC = 0.509) (D), or the ratio of Fila-αS/Total-αS (AUC = 0.74) (F) was computed. Dotted line: random classifier.

**Figure 4.**
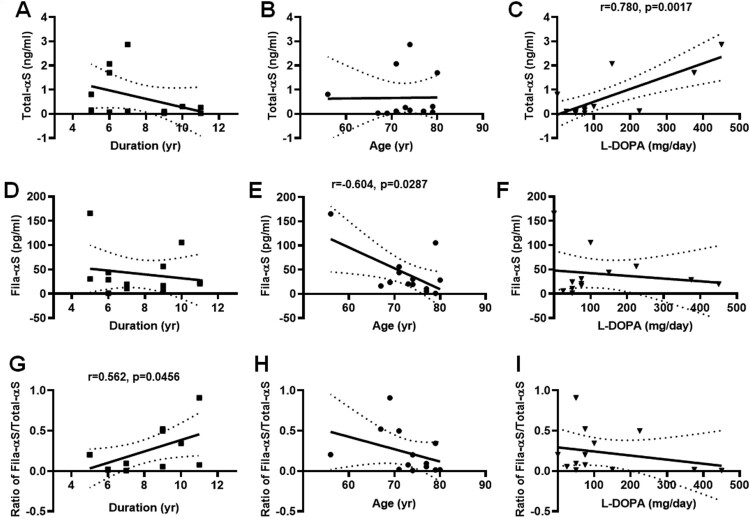
Correlations of urinary α-syn with various parameters of PD. The levels of Total-αS in the urine compared with the on-set duration (A), age (B), and L-DOPA dosage (C). The levels of Fila-αS in the urine compared with the on-set duration (D), age (E), and L-DOPA (F). The ratio of Fila-αS/Total-αS estimated, and its correlation with the on-set duration (G), age (H), and the administration dose of L-DOPA determined (I). All XY correlations was computed by Pearson correlation coefficient (PD = 13).

For the validation of α-syn release into biofluids, we collected human urine samples from 13 PD patients and 8 non-PD subjects. Our previous study demonstrated the presence of α-syn in the urine and distinct changes between two types of α-syn oligomers and total α-syn in urine. In this study, we attempted to compare the fibrillar oligomer α-syn to total α-syn in urine using our ELISA. Then, we estimated our ELISA analyses via receiver operating characteristic (ROC) curve and the area under the ROC curve (AUC). Total-αS levels were significantly lower in PD patients than in non-PD subjects (Figure 3(A)), and the AUC of ROC curve from Total-αS levels was 0.750 (Figure 3(B)). The Fila-αS levels showed no differences (Figure 3(C)), and its ROC curve closed to the dotted line of random classifier (AUC = 0.509) (Figure 3(D)). The ratio of Fila-αS/Total-αS exhibited a slight increase in PD patients than non-PD subjects (Figure 3(E)), however the ROC curve showed a substantial reliability (AUC = 0.740) (Figure 3(F)). In addition, Total-αS levels was increased in line with the prescribed L-DOPA dosage (Figure 4(C)); however, the on-set duration or age were not correlated (Figure 4(A,B)). Fila-αS showed no significant correlations with on-set duration or L-DOPA dose (Figure 4(D,F)), with the exception of decreased Fila-αS with the PD patients’ age (Figure 4(E)). The ratio of Fila-αS/Total-αS exhibited a significant increasing correlation curve with on-set duration (Figure 4(G)), while its correlation with age or L-DOPA dosage showed similar patterns to those of Fila-αS (Figure 4(H,I)). These results suggested that the various PD-representing parameters would not be uniform when compared with the levels of distinct α-syn species.

## Discussion

The release of α-syn has been studied by numerous groups, and most reports revealed that α-syn secretion was triggered by the accumulation of α-syn in the neuronal cells (Fussi et al. 2018; Hijaz and Volpicelli-Daley 2020). G2019S LRRK2 mutant is regarded to induce accumulation of α-syn, thereby accelerating the aggregation of α-syn in the cytoplasmic vesicles or autophagosome/lysosome (Schapansky et al. 2018). Moreover, several studies have demonstrated that the expression of G2019S LRRK2 in neuronal cells provokes α-syn secretion (Bae et al. 2018; Bieri et al. 2019). Similar to previous studies, the accumulation of Total- or Fila-αS in dSH was increased by the ectopic expression of G2019S LRRK2 (Figure 1(B–C)). The transient expression of myc-G2019S in dSH aggravated the accumulation of Total-αS such that the increase of the α-syn aggregation in cytoplasm could elevate the levels of intracellular Fila-αS. Tissues of SN from non-PD or PD patients showed a LRRK2-dependent Total-αS increase (Figure 2(D,E)); this might be owing to the accumulation of α-syn as a result of LRRK2 activity. The detection of Total-αS is regarding to detecting various types of α-syn, including monomers, dimers, trimers, tetramers, or tiny oligomers, even fibrillar α-syn oligomers. Despite the ambiguous understanding of the effect of α-syn fibrils on PD pathogenesis, it has been known that α-syn fibrils represent a non-toxic form of α-syn oligomers (Zhang et al. 2020). However, fibrillar conformation is critical for the prion-like propagation of α-syn via cell-to-cell transmission (Freundt et al. 2012; Wu et al. 2019). Hence, the LRRK2-dependent Total-αS increase might indicate that other oligomeric α-syn types accumulate to a greater extent in the SN of PD patients than non-PD controls, which in turn, could promote the progression of PD. Simultaneously, fibrillar α-syn might be constantly released during the progression of PD; thereby increasing α-syn propagation. Although the ratio of Fila-αS/Total-αS was slightly increased, this was not significant (Figure 2(C)); thus, we should expand the samples of human SN lysate to concrete their change between PD and non-PD.

Our previous study showed a significant decrease in Fila-αS in urines from PD patients compared with that from non-PD subjects (Nam et al. 2020). In this study, the Fila-αS levels in PD urine were not higher than those in the non-PD urine (Figure 2(C)), but the ratio of Fila-αS/Total-αS was slightly increased in PD patients (Figure 2(E)). This might be because of the differences in the urine sampling process because we increased the speed of centrifugation at the pre-concentration step to spin down the protein adducts and salt crystals that would have interfered with the ELISA. Besides, this methodological alteration would have led to the enhancement of ELISA absorbance, so the detected scale of Total-αS and Fila-αS levels were increased than our previous study. The levels of Total-αS were decreased in PD urines (Figure 3(A)), and previous studies reported that the levels of total α-syn in plasma were lower in PD patients than in controls (Ishii et al. 2015). Intriguingly, the dosage of L-DOPA in PD patients has shown a positive correlation curve with Total-αS levels. Indeed, a previous study revealed that L-DOPA treatment increased the aggregation of α-syn and its release from dSH cells (Lee et al. 2011). Recently, oxidation of dopamine promoted a loss of lysosomal activity and mitochondrial function, thereby increasing α-syn accumulation (Burbulla et al. 2017). Although it has not yet been elucidated whether L-DOPA or α-syn represent the cause or result, L-DOPA administration might affect the release of α-syn via deteriorating the accumulation of α-syn. Since the ROC curve of Total-αS levels exhibited a reliable AUC value, the decrease in total urinary α-syn may be one of PD biomarker. However, the oligomeric state of α-syn could be dynamically changed by sampling or storage processes; thereby accelerating the dissociation oligomeric α-syn to monomer α-syn or the oligomerization. These led us arduous to reproduce α-syn ELISA analyses, and we assume that it is another reason why our first- and second-order results are inconsistent.

We observed a decline in the correlation curve of Fila-αS levels with the age of PD patients (Figure 4(E)). A previous study demonstrated that the levels of total α-syn in plasma declined with aging of healthy subjects (Koehler et al. 2015). We hypothesized that the urinary α-syn might originate from α-syn in blood because (1) renal dysfunction was reported as a risk factor in PD patients and (2) incomparable high levels of urinary α-syn were detected in proteinuria patients in our preliminary research (data not shown). Although we did not observe any significance between aging and the levels of α-syn in non-PD subjects (Supplementary Figure 1), aging might affect the dynamics of α-syn secretion in PD patients.

The ratio of Fila-αS/Total-αS increased with the on-set duration of PD patients and showed an ascending correlation curve (Figure 4(G)). The correlation curves of age or L-DOPA administration compared with the ratio of Fila-αS/Total-αS (Figure 4(E,F)) showed a similar curve pattern to that of Fila-αS levels along with age or L-DOPA (Figure 4(H,I)). Because (1) the individual differences of on-set age across PD patients are unpredictable and (2) ROC curve from the ratio of Fila Fila-αS/Total-αS showed a reliable AUC value, we expected that the on-set duration of PD patients increased along with the ratio of Fila-αS/Total-αS can be considered as a diagnostic tool for PD. However, we need a follow-up study with larger samples.

We analyzed the correlations of α-syn in urine between the urinary oxidized DJ-1 (OxiDJ-1) which was reported as a feasible marker of PD in our previous study (Jang et al. 2018), and urinary LRRK2 levels which were reported as a potential marker of PD in previous studies (Fraser, Moehle, et al. 2016; Fraser, Rawlins, et al. 2016). However, we were not able to find any relation between OxiDJ-1 and urinary α-syn levels (data not shown). The measurement of urinary LRRK2 levels using western blot was impossible because most of PD patients or non-PD subjects harbored undetectable levels of LRRK2 (Supplementary Figure 2); thus, we believe that methodological improvements would be required for the detection of urinary LRRK2.

In this study, we developed an ELISA for detecting urinary α-syn and demonstrated the changes in Total-αS, Fila-αS, or their ratio using dSH, SN tissues from human subjects, and patients’ urine. However, the results across cells, post-mortem tissues, and fresh biofluids are not consistent, but the ratio of Fila-αS/Total-αS shows promising results. To verify the validity of our findings, the expansion of PD patients and non-PD subjects is necessary in further studies, and a longitudinal study of PD from the healthy condition to post-mortem may be worthwhile in the future. In conclusion, it is important to compare and use various PD-parameters when developing molecular biological diagnostic tools based on our novel findings. Through this process, approaches to the appropriate diagnosis can be improved.

## Supplementary Material

Supplemental Material

## Data Availability

The datasets generated during and/or analyzed during the current study are available from the corresponding author on reasonable request.
