# Supplementary material for: Analysis of α-synuclein levels related to LRRK2 kinase activity: from substantia nigra to urine of patients with Parkinson’s disease
Source: Anim Cells Syst (Seoul). 2021 Feb 17;25(1):28–36. doi: 10.1080/19768354.2021.1883735 (PMC7935126; doi:10.1080/19768354.2021.1883735)
Supplement: Supplemental Material [file TACS_A_1883735_SM8764.pdf]

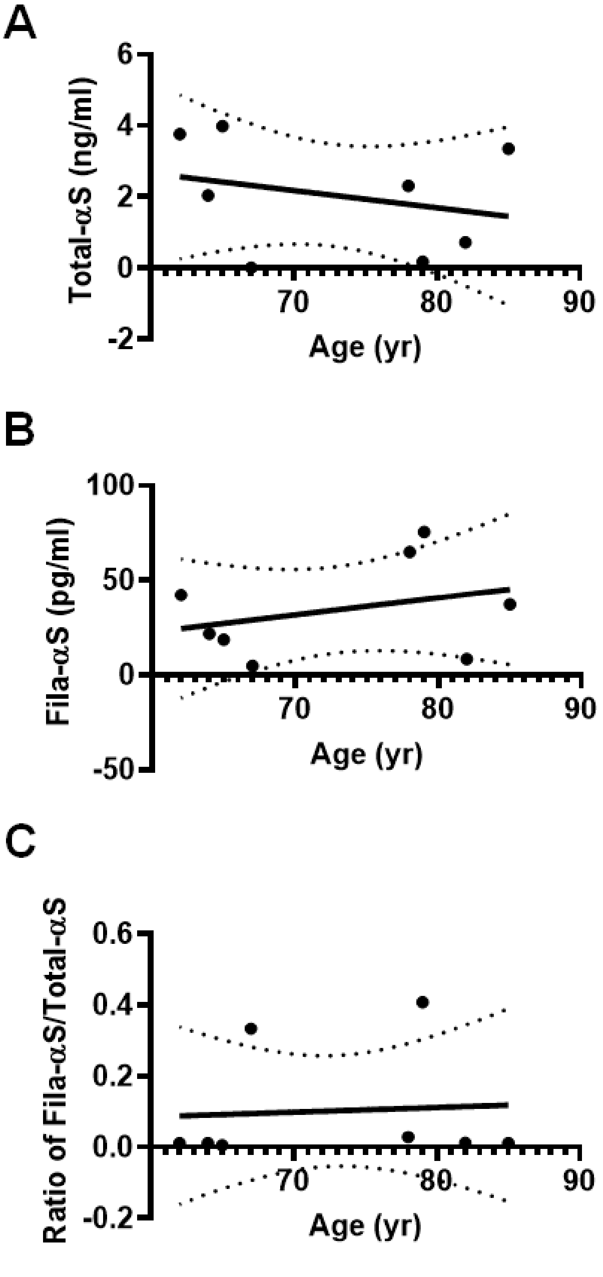

Supplementary Figure 1. Correlation between the age of non-PD subjects and the levels of urinary α-syn. The levels of Total-αS (A), Fila-αS (B), and the ratio of Fila-αS/Total-αS (C) compared with respect to the age of the 8 non-PD subjects.

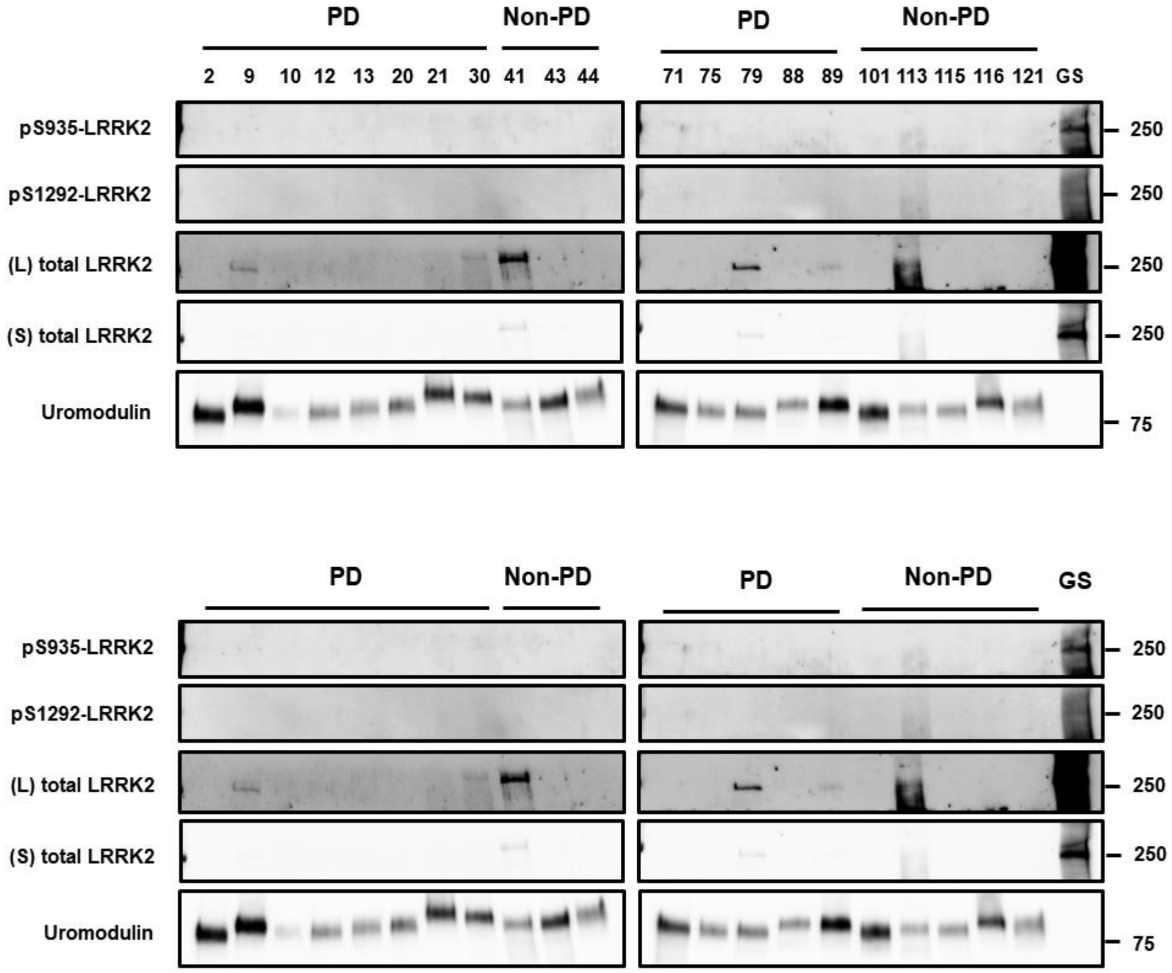

Supplementary Figure 3. Detection of LRRK2 in human urines. For western blot, 20 μl of urine samples used. Mouse monoclonal anti-uromodulin antibody (Santa Cruz, sc-271022, 1:500) used as the loading control for urine samples. dSH-expressing ectopic G2019S LRRK2 (GS) used as the positive control for LRRK2, pS1292-LRRK2 (1:300), and phosphorylation at the S935 site (pS935-LRRK2) (Anti-LRRK2 [phosphor S935] antibody, Abcam, ab133450, 1:500) and a negative control of uromodulin (non-PD = 8, PD = 13). L: long exposure, S: short exposure
